# Molecular Docking Evaluation of (*E*)-5-arylidene-2-thioxothiazolidin-4-one Derivatives as Selective Bacterial Adenylate Kinase Inhibitors

**DOI:** 10.3390/molecules23051076

**Published:** 2018-05-03

**Authors:** Mihaela Ileana Ionescu, Ovidiu Oniga

**Affiliations:** 1 Department of Microbiology, Iuliu Hațieganu University of Medicine and Pharmacy, 400349 Cluj-Napoca, Romania; 2 Department of Microbiology, County Emergency Clinical Hospital, 400006 Cluj-Napoca, Romania; 3 Department of Pharmaceutical Chemistry, Faculty of Pharmacy, Iuliu Hațieganu University of Medicine and Pharmacy, 400012 Cluj-Napoca, Romania; onigao65@yahoo.com

**Keywords:** adenylate kinase, thiazolidine, molecular docking, inhibitory activity

## Abstract

Multi-drug resistant microorganism infections with emerging problems that require not only a prevention strategy, but also the development of new inhibitory compounds. Six previously synthesized 5-arylidene-2-thioxothiazolidin-4-one derivatives **1a**–**f**, were screened for inhibitory activity on adenylate kinases of different origins by molecular docking. The compounds **1c** and **1d** were the most efficient inhibitors of bacterial and some archean adenylate kinases. Hydrogen bond interactions were observed with the residues belonging to the ATP binding site. Moreover human adenylate kinases are poor targets, suggesting that this selectivity offers promising prospectives for refining the structure of our compounds.

## 1. Introduction

Sulfur is a non-metal widespread in Nature both as the pure element or as sulfide or sulfate minerals, while sulfur dioxide (SO_2_) and hydrogen sulfide (H_2_S) are abundantly found in the gases emitted by volcanoes. The ancient people, good observers of the world, not only discovered the healing properties of sulfurous springs, but in Roman mythology, along myriads of deities, there was a goddess Mephitis responsible for the unpleasant smell of these environments. Sulfur-containing compounds are widely found in Nature and climatic changes, which have occurred during billions of years, greatly influence the chemical composition of soil. Consequently, all living things must have evolved in parallel with inorganic and organic compounds found in each specific ecologic niche [1,2,3]. The sulfur is essential for human health and sulfur-containing compounds could influence human health in both senses—beneficially or related with the most diverse pathologies [4,5].

The pharmaceutical industry has developed a huge panel of sulfur-containing drugs with anti-rheumatic, oncological or anti-inflammatory effects [6,7]. New compounds with antibacterial effect are continuously being developed [8,9]. Nowadays there is a great interest in finding natural compounds with therapeutic effect, such garlic extract [10]. Although it seems there is a low interest towards synthesis of new sulfur-containing heterocycles—only 382 results, on 17 March 2018, when searching “sulfur-containing AND heterocycles” on Clarivate Web of Science—the biological implications of some of them could reveal unexpected applications. Focusing on a “thiazolidine” search, among 2661 results, after refining search with the query “clinical trials” only 22 papers were selected. While the health care professionals are always willing to start clinical trials, the new sulfur-containing heterocycles remains in the synthesis and characterization phase. Finding new compounds implies accurate target identification. Thiazolidine derivatives show antibacterial or antifungal activity, inhibiting the most diverse structural or functional enzymes. A recent experimental study showed that some 1,2,4-triazolothiazolidin-4-one derivatives perturb the cellular integrity by inhibition of an enzyme involved in peptidoglycan synthesis— UDP-*N*-acetylenolpyruvoylglucosamine reductase (MurB) [11]. Other authors identified *Mycobacterium tuberculosis* pantothenate synthetase as a target for thiazolidine derivatives [12]. Thiazolidine derivatives definitely, have interesting features that are not sufficiently explored, but the real concern is related to their side effects on human enzymes.

Enzymes involved in energetic metabolism, as nucleoside monophosphate kinases, have two main characteristics—ubiquity and cruciality for life [13,14,15]. Adenylate kinase (AK)—ATP:AMP phosphotransferase; EC 2.7.3.4—catalyzes the reversible transfer of the phosphate group between ATP and AMP (Mg^2+^ ATP + AMP = Mg^2+^ ADP + ADP). Some isoenzymes—AK3 and AK4, also named AK3-like—are GTP:AMP phosphotransferase; EC 2.7.4.10 [16,17,18]. It is a usually monomeric enzyme that has been chosen as a model in many kinetic studies, its conformational changing during catalytic reactions being revealed by crystallographic studies of the unliganded enzyme or of the enzyme in complex with the substrate analog Ap5A [19]. It has a three-domain structure which undergoes closure through a hinge mechanism. The large central domain, named CORE domain, is flanked by two small domains—the AMP binding domain (AMPbd) and the ATP-binding domain (ATPlid) or LID domain [20]. The latter closes over the site of phosphoryl transfer upon ATP binding [21], resulting in a so-called closed conformation [22,23]. The conformational rearrangement process of the ATPlid domain and, to a smaller measure, of the AMPbd domain is not clearly understood. Experimental studies show that the flexible domains fold in a noncooperative manner with respect of the CORE domain [24]. This conformational pathway is not a steep one. Between the open and the closed conformations, there are also intermediate states. Coarse-grained molecular dynamics simulation studies provide insight into the residues involved in structural fluctuation in the open, closed, and intermediate forms [25]. The large-conformational movement of the ATPlid, at the beginning of catalytic process, has a much more complex role than just proper alignment of the substrates in the active sites. It is demonstrated that the catalytic selectivity for ATP over GTP lies on the ability of the same residues—Arg 118, Arg 123, and Arg 167—to influence the positive selection of ATP and the negative selection of GTP. Therefore, energy homeostasis is maintained by reducing the depletion of the cellular GTP pool [26]. AK, a ubiquitous enzyme, shows remarkable adaptation over billions of years of evolution from a hot environment to a cooler temperature, the central CORE domain determining the melting temperature of the AKs. [24]. Therefore it is not a surprise that it is present in all three domains of life—Bacteria, Eukarya, and Archaea. Until now, nine AK isoforms were identified.in eukaryotes distributed in different intracellular compartments. While AKs isoenzymes 1, 5, 7 and 8 are cytosolic, AK6 and AK 9 are found in the nucleus. AK2, AK3, and AK4 are mitochondrial isoenzymes—AK2 being located in the intermembrane space [27,28]. Some studies demonstrated the role of AK of *Burkholderia pyrrocinia* in expressing its antifungal activity [29]. It is well established that elemental sulfur is a specific inhibitor of AN activity from skeletal muscle, erythrocytes, and cardiac tissue [30] and sulfhydryl compounds promote conformational changes of AKs, a feature which could be exploited in controlling the inhibitory activity of other compounds [31].

All these facts presented above led us to consider AK as an interesting choice for testing new sulfur-containing heterocycles. In a previous work, a series of 5-arylidene-2-thioxothiazolidin-4-one derivatives demonstrated inhibitory activity on AKs from *Escherichia coli* and *Streptococcus pneumonia*. All derivatives were initially tested at one concentration (0.2 mM), then the best inhibitor—herein named **1d**—was selected for further kinetic analysis. The I_50_ values (the inhibitor concentration that leads to 50% activity inhibition) were 0.067 mM for *E. coli* AK and 0.065 mM for *S. pneumoniae*, respectively [32,33]. We assumed that the binding site of this derivative is the same for AKs of both origin. To decipher the binding of this series of 5-arylidene-2-thioxothiazolidin-4-one derivatives, a different approach was attempted. Even though considered an empirical method, molecular docking is a useful tool to gain insights into ligand-enzyme interactions and for screening AKs of different origin, mostly comparing AKs of bacterial pathogens with their human counterparts.

## 2. Results

First, the raw data—binding energy ∆G (kcal/mol) and inhibition constant Ki (µM) of the best conformation after molecular docking—were analyzed by statistical tests. We attempt to organize the data started by the hypothesis that there is no difference between inhibitory activities of the derivatives **1a**–**f** for AKs of bacterial or human origin, but, before that, we have to find out if derivatives **1a**–**f** interact with the active site residues of AKs. Therefore, the 3D structures of unliganded AKs—open conformation—were compared with their closed-conformation counterparts. In Table 1 we selected the binding energy and the inhibition constant values only for the AKs that have 3D structured in the Protein Data Bank (PDB) in both conformations. In our previous experimental studies [32,33], an extra compound was tested—C_10_H_6_FNOS_2_, with the substituent R = 3F—but we failed to docked it. We were also unable to perform the docking of 4NTZ with **1b** and **1c**, 1Z83 with **1d** and **1f**, and 2AR7 with **1b**, **1d** and **1e**, respectively.

In Table S1 (Supplementary Material) we compare the overall inhibitory activity of the derivatives **1a**–**f** against Bacteria, Eukarya (human), and Archaea AKs. Since only two archaean AKs, were included in the present study, data obtained after molecular docking was not statistically analyzed by Student’s *t*-test. However, we performed a statistical analysis of the binding energy and the inhibition constant values of all AKs in closed conformation to have a global picture of the results. Statistical analysis of the data reveals that there is enough evidence to indicate that the means are not the same. Because the One-Way ANOVA test relies on the variability between the populations, a second statistical test was performed. The Kruskal–Wallis H test confirmed the results obtained by the previous test. Then, after a detailed analysis of the raw data and the statistical interpretation of molecular docking parameters, we attempted to understand the intrinsic mechanisms of AK inhibition by derivatives **1a**–**f**. From the Bacteria domain we selected the AKs from the mesophilic *Escherichia coli* (3HPQ), *Streptococcus pneumoniae* (4NU0) and *Bacillus subtilis* (1P3J), the thermophilic *Bacillus stearothermophilus* (1ZIP), the hypertermophilic *Aquifex aeolicus* (2RGX), and the psychrophilic *Bacillus globisporus* (1S3G). Five isoforms of human origin were found as having the 3D structure co-crystallized with inhibitors or similar substrates—the AK1 (1Z83 and 2C95), AK2 (2C9Y), AK4 (2BBW) and AK5 (2BWJ-chain B). Only two AKs from archean species were selected—the mesophilic *Methanococcus voltae* (1KHT/chain B) and hypertermophilic *Sulfolobus acidocaldarius* (1NKS/chain F). Table 2 shows the statistical analysis of inhibitory activity of the derivatives **1a**–**f** against bacterial and human AKs in closed-conformations—the ∆G (kcal/mol) and Ki (µM) data are shown in the Table S1 as mean and standard deviation. We noted that the most significant differences between inhibitory activity against AKs of bacterial and human origin are for the compound **1c**, therefore we have focused on observing the residues involved in binding.this compound. Since these observations are not consistent with our previous experimental studies, when the **1d** derivative demonstrated the best inhibitory activity against *E. coli* and *S. pneumoniae*, a thorough analysis of the raw docking data was performed.

**Figure 1:**
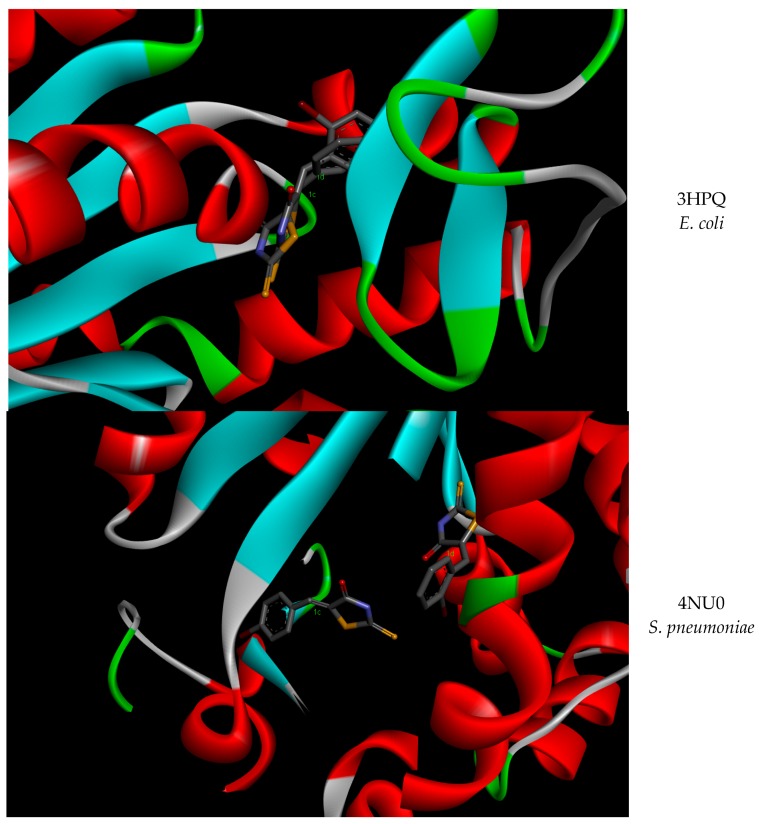
The interactions of the **1c** and **1d** derivatives with *E. coli* and *S. pneumoniae* AKs.

In Table 3 and Figure 1 a comparison of the inhibitory activity of the derivatives **1c** and **1d** demonstrate that their interactions are nor the same, although the experimental data suggest a similar inhibitory mechanism. However, for *E. coli* AK, **1c** and **1d** interactions are comparable, as shown in the Tables S2 and S3.

A deeper insight of ligand-protein interactions for the compounds **1c** and **1d** reveals more evidence of its efficiency in distinct inhibition of bacterial and some archean AKs. In Tables S2 and S3, Figures S1 and S2 we show the atomic interactions of the best conformations obtained by molecular docking of compounds **1c** and **1d** with different AKs. The AKs’ interacting residues with AMP, ATP, or ADP were provided the Protein Features module of the PDB. Hydrogen bond interactions were observed with the residues from the ATPlid for bacterial AKs with a subtle exception for *S. pneumoniae* AK which forms a hydrogen bond with a residue belonging to AMPbd. The hypertermophilic bacteria *A. aeolicus*, and the mesophilic archeon *M. voltae* form hydrogen bonds with AMPbd. Also, the psychrophilic *B. globisporus* forms only one hydrogen bound with an ATPlid residue. The hyperthermophilic archeon *S. acidocaldarius* forms only two hydrogen bonds with residues which do not belong to AMPbd or ADPbd—the substrates which were co-crystallyzed with 1NKS. Finally, *B. stearothermophilus* and *M. voltae* interact with residues which do not belong to the AK active site.

**Figure 2:**
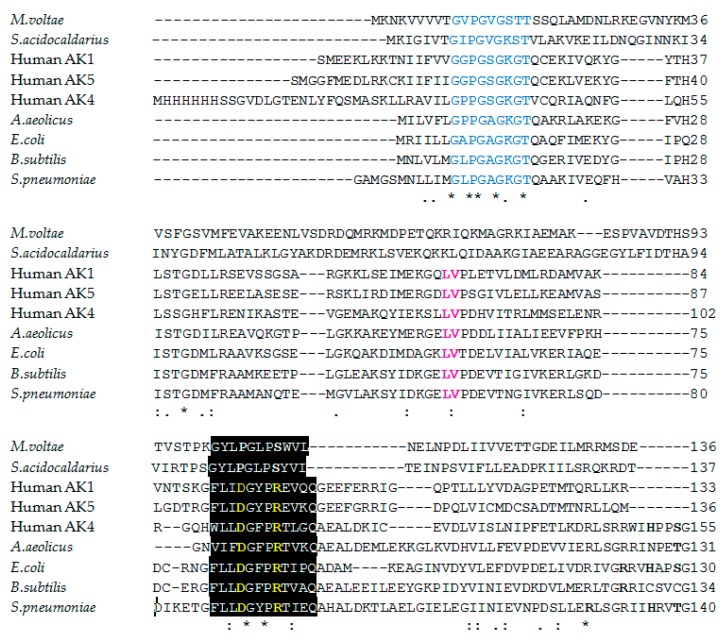
Multiple sequence alignment of the AKs’ sequences. Marked in blue are the residues belonging to the Walker A motif—phosphate-binding loop or P-loop; in the black background was selected the consensus sequence [LIVMFYWCA]-LIVMFYW] (2) -D-G-[FYI]-P-R-X (3) -[NQ] and in yellow were selected the residues strictly conserved in the adenylate kinase family; with pink were selected the Lys and Val residues—well conserved in AKs. Multiple sequence alignment was performed by the Clustal Omega program (https://www.ebi.ac.uk/Tools/msa/clustalo). * (asterisk) indicates positions which have a single, fully conserved amino acid residue; : (colon) indicates conservation between groups of strongly similar properties as following—roughly equivalent to scoring >0.5 in the Gonnet PAM 250 matrix: STA, NEQK, NHQK, NDEQ, QHRK, MILV, MILF, HY, FYW; . (period) indicates conservation between groups of weakly similar properties as following—roughly equivalent to scoring =<0.5 and >0 in the Gonnet PAM 250 matrix: CSA, ATV, SAG, STNK, STPA, SGND, SNDEQK, NDEQHK, NEQHRK, FVLIM, HFY

In Figure 2 multiple sequence alignment of nine sequences show that the residues involved substrate binding are well conserved among AKs of all domain of life, with notable differences among the archean enzymes where nine sequences were aligned. 

## 3. Discussion

The discovery of new antibacterial compounds is a great challenge because microorganisms are so versatile that they inevitably develop smart resistance mechanisms. Before understanding the subtle mechanisms of inhibitory activity of new compounds against an unusual target like AK, it is essential to accurately identify the targets. Otherwise, we will be stuck in time-consuming and costly experiments without valuable results. AK is an interesting enzyme that still raises the interest of many research teams even though its structure and kinetic properties are well known. When it comes to starting a new project, such as synthesis and testing the inhibitory activity of new compounds, it is better to use a well characterized target. In a previous work, we discovered a good inhibitory activity of some thiazolidine derivatives against *Escherichia coli* [32] and *Streptococcus pneumonia* AKs [33]. Because the derivatives **1a**–**f** are soluble in dimethylformamide (DMF) the inhibitory activity of these compounds against AK was possible only by performing by a 2,4-dinitrophenylhydrazine colorimetric assay, not by the coupled spectrophotometric assay, a more reliable method for activity assay of AK [19]. Therefore, six 5-arylidene-2-thioxothiazolidin-4-one derivatives **1a**–**f** were further explored to predict their binding interactions by molecular docking. The results confirm that compound **1d** C_10_H_12_BrNOS_2_ (R = 1 Br) inhibits bacterial AKs targeting the ATPlid residues of the active site. More, comparing the cumulative docking data for AKs of bacterial and human origin, we could reject the null hypothesis that there are no differences between the two groups (Table S1 and Table 2), therefore derivatives **1a**–**f** are worth a deeper analyses of their inhibitory activity against bacterial AKs. Statistical tools are useful as the first step of designing a study, providing valuable information about the relevance of disparate data. After the preliminary statistical analysis, we continued the molecular docking study, which is considered an empirical method, but providing some clues about interacting residues. During conformational changes, water molecules removal from the active sites prevent nonproductive side reactions, therefore, it is relevant to observe the influence of water molecules in inhibitory binding [26]. In the present study, we considered AKs as rigid receptors and the inhibitors as flexible ligands. The molecular docking should be confirmed with experimental data, but our goal was first was first to advance the analysis of the previous experimental data with an inexpensive method, then to improve the structures of the best thiazolidine derivative for future studies.

For the derivatives **1a**–**f**, we can compare the docking data with experimental data only for *E. coli* and *S. pneumoniae* AKs, but these results are consistent and we can assume that the docking data are representative for the rest of the tested AKs. The most concerning issue is about the inhibition of the human AKs. Without experimental data, we can only draw a preliminary conclusion on their interaction with derivatives **1a**–**f**. Our results are quite encouraging in designing a future experimental study about inhibition of human AKs by derivatives **1a**–**f**. Here is a major limitation due to the low solubility of the **1a**–**f** derivatives. Obviously, the colorimetric method previously used could be applied, but our results are promising for designing new thiazolidine compounds with similar structures, but with a better solubility in Tris buffer in order to perform a more reliable kinetic study.

The main limitation of accurate kinetic studies of the inhibitory activity of the vast majority of new synthetic compounds is their low water solubility, although promising results exist [19]. Thus molecular docking is an appropriate suitable alternative approach for deciphering the molecular mechanisms of ligand binding, although there are limitations in the availability of 3D structures in public databases. However, the present study demonstrates that AK is a valuable target for certain thiazolidine derivatives and we will consider screening other enzymes and designing new similar compounds.

Another interesting perspective is related to the pollution of the environment with synthetic compounds and the way they can influence the species of microorganisms in certain ecologic niches. Sulfur-containing compounds are found to be industrial waste which, in concert with other compounds, influences environmental microorganisms. Perhaps only ecologists are interested in the dynamics of environmental microorganisms, but the subject is not a minor one. Suffice it to say that the soil environment directly influences the quality of drinking water. These problems are not difficult to solve, but we are far from understanding them. We can only try to imagine what happens when some synthetic compounds are spread in Nature. This is the main reason of including AKs of archeal origin and AKs from bacterial species from extreme environments. AKs from the Archea and extremophile species interacts with AMPbd residues (Tables S2 and S3) contrary to their mesophylic counterparts, which interact with ATP-binding residues.

Taken together, AK is an interesting enzyme not only for the development of new thiazolidine derivatives or other sulfur-containing compounds but can play a crucial role in understanding the sophisticated mechanisms developed by microorganisms or eukaryots in maintaining energy homeostasis.

## 4. Materials and Methods

The methodology included: (1) ligand preparation for molecular docking; (2) acquisition of 3D structures of AKs and preparing them as receptors for molecular docking; (3) molecular docking protocol; and (4) statistical analysis.

### 4.1. Ligand Preparation

**Figure 3:**
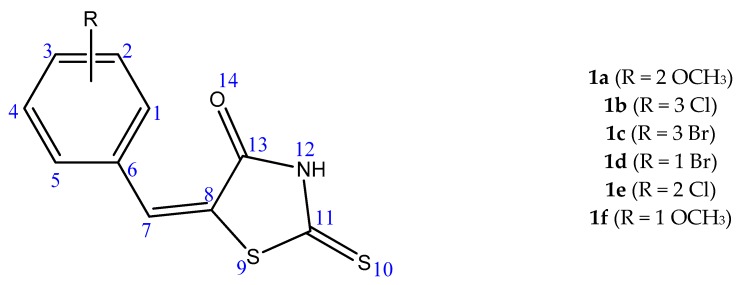
The structure of the 5-arylidene-2-thioxothiazolidin-4-one derivatives **1a**–**f**, R = various substituents.

In the Figure 3 is represented the structure of derivatives **1a**–**f** used as ligands in our protein docking experiments and their chemical characteristics are given in Table S4. Their synthesis was previously described in another paper [34]. Briefly they were obtained by Knoevenagel condensation between various substituted aryl aldehydes and thiazolidin-4-on-2-thione, by refluxing in glacial acetic acid, in the presence of anhydrous sodium acetate. The ChemDraw 10.0 program was used for drawing, displaying and characterizing chemical structures. The optimized 3D structures were saved as pdb files for molecular docking preparation by AutoDockTools.

### 4.2. Adenylate Kinases Structure Acquisition and Preparation as Receptors

The main characteristics of the AKs included in the present study are presented in the Table S5. In the present study we considered AKs from the three domains of life—Archaea, Bacteria, and Eukarya [23,35,36,37,38,39,40,41]. For a better comparative analysis, AKs from the same species in closed conformation—co-crystallized with Ap5A, AMP, or ATP—and in open conformation were first collected. The enzymes were selected without mutations in their sequences. First, the AKs’ X-ray 3D structures were downloaded from the Protein Data Bank PDB (https://www.rcsb.org/structure). From the 3D structure, all solvent molecules were removed and the substrates when proteins were crystallized in closed conformation. For the multimeric AKs structures, we first chose the chain crystallized with substrate or inhibitor then the chain crystallized without substrate or inhibitor. When all chains are alike, the A chain was selected. The Dassault Systèmes BIOVIA—Discovery Studio Modeling Environment (Release 2017, Dassault Systèmes, San Diego, CA, USA, 2016; http://accelrys.com)—allows quick preparations of the protein structures. After removing water molecules, and other substrates or inhibitors, the AKs were saved as pdb files for molecular docking preparation which involved the addition of non-polar hydrogen and of Kollman charges, computed by AutoDockTools.

### 4.3. Molecular Docking Protocol

Although there are many programs for protein-ligand docking, we concluded that the AutoDock4.2 program, distributed as open source under a GPL license, perfectly accomplished the requirements for designing the present study (http://autodock.scripps.edu) [42,43]. For non-experts, BIOVIA is a friendly program for further interpretations of docking data on Windows platform. It allows the observation of the best docking conformation, permits one to visualize the protein-ligand 2D diagram of the interacting atoms, and offers protein-ligand complex 3D molecular visualization tools. Ligand flexibility was assigned in the following steps—detect the root that minimizes the size of the largest branch, set the number of torsions in the interval 1–6, then choose aromaticity criterion at 7.5′. Herein, we used an algorithm based on a rigid protein structure of AKs and the Lamarckian Genetic Algorithm (LGA), with a maximum of 2,500,000 energy evaluations as searching parameter. Otherwise, the search parameters and docking parameters were used as defaults. The Cygwin DLL (cygwin1.dll) terminal, which provides functionality similar to a Linux distribution on Windows, was necessary for running the molecular docking scripts.

### 4.4. Statistical Analysis

Data were expressed as mean ± standard deviation. The *p*-value was calculated with the Student’s *t*-test—two-tailed hypothesis at 0.05 significance level. For comparing more than two samples, the One-Way ANOVA test and Kruskal–Wallis H test were performed.

## Figures and Tables

**Table 1:** The binding energies (kcal/mol) and inhibition constants (µM) of the unliganded AKs or of the enzyme in complex with the substrates or substrate analogs.

Strain	AK Conformation (PDB ID ^1^)	∆G ^2^ (kcal/mol) Mean (±SD ^3^)	Ki ^4^ (µM) Mean (±SD)	*p*-Value	
				∆G (kcal/mol)	Ki (µM)
*E. coli*	O ^5a^ (4AKE)	−5.26 (±1.7)	51.32 (±27.53)	0.020285	0.002378
	C ^5b^ (1HPQ)	−7.19 (±0.24)	5.75 (±2.59)		
*S. pneumoniae*	O (4NTZ)	−5.59 (±2.48)	18.12 (±14.01)	0.2097	0.177056
	C (4NU0)	−6.97 (±0.42)	9.23 (±4.55)		
*A.* *aeolicus*	O (2RH5)	−5.81 (±0.32)	59.53 (±22.31)	0.000898	0.000854
	C (2RGX)	−6.77 (±0.4)	13.21 (±9.37)		
*S. acidocaldarius* ^6^	O (1NKS/chain A)	−5.87 (±0.09)	50.61 (±7.75)	0.000975	0.000341
	O/C (1NKS/Chain C)	−5.91 (±.44)	56.04 (±32.03)		
	C (1NKS/Chains F)	−6.46 (±0.31)	34.77 (±14.19)		
*M. voltae*	O (1KHT/Chain A)	−5.69 (±0.35)	81.06 (±65.92)	0.000054	0.024538
	C (1KHT/Chain B)	−6.88 (±0.26)	9.73 (±4.31)		
Human–AK4	O (2AR7)	−6.11 (±0.22)	34.48 (±13.15)	0.056913	0.034455
	C (BBW)	−6.52 (±0.27)	17.85 (±6.62)		
Human–AK5	O (2BWJ/Chain A)	−6.41 (±0.27)	22.19 (±13.11)	0.010154	0.020725
	C (2BWJ/Chain B)	−5.77 (±0.41)	70.03 (±40.66)		

^1^ Protein Data Bank Accession number; ^2^ Binding Energy; ^3^ Standard Deviation; ^4^ Inhibition Constant; ^5a^ O = Open conformation of AK, ^5b^ C = Closed-conformation of AK; ^6^ *p*-value for 1NKS/chain A and 1NKS/Chains F comparison.

**Table 2:** Statistical analysis of the inhibitory activity of the derivatives **1a**–**f** against closed-conformation of bacterial AKs (2RGX, 3HPQ, 4NU0, 1P3J, 1ZIP, 1S3G) and eukaryotic AKs (1Z83, 2C95, 2C9Y, 2BBW, 2BWJ/chain B).

Derivative	^1^ ∆G/Ki	*t*-Value	*p*-Value	^2^ ∆µ	^3^ CI 95%
**1a**	∆G	0.09867	0.923565	0.0357	−0.7821 ≤ ∆µ ≥ 0.8534
	Ki	1.06499	0.314617	18.4687	−20.7608 ≤ ∆µ ≥ 57.6981
**1b**	∆G	3.84529	0.003935	0.933	0.3841 ≤ ∆µ ≥ 1.4819
	Ki	2.4127	0.039076	46.6247	2.9091 ≤ ∆µ ≥ 90.3402
**1c**	∆G	4.49226	0.001506	0.958	0.4756 ≤ ∆µ ≥ 1.4404
	Ki	2.2898	0.04779	33.761	0.4075 ≤ ∆µ ≥ 67.1145
**1d**	∆G	1.8825	0.09634	0.66	−0.1485 ≤ ∆µ ≥ 1.4685
	Ki	1.87666	0.097405	21.2967	−4.8723 ≤ ∆µ ≥ 47.4
**1e**	∆G	2.31486	0.04587	0.8183	0.0186 ≤ ∆µ ≥ 1.6180
	Ki	1.53788	0.158459	58.1810	−27.4010 ≤ ∆µ ≥ 143.7630
**1f**	∆G	1.08505	0.309513	0.325	−0.3657 ≤ ∆µ ≥1.0157
	Ki	1.21529	0.258901	27.4042	−24.5951 ≤ ∆µ ≥ 79.4034

^1^ ∆G (kcal/mol), Ki (µM); ^2^ the difference between eukaryotic mean and bacterial mean; ^3^ 95% confidence interval of the difference between the eukaryotic mean and bacterial mean.

**Table 3:** Analysis of the inhibitory activity of the derivatives **1c** and **1d** against open and closed-conformations of *E. coli* and *S. pneumoniae* AKs.

Strain	PDB ID	1c		1d	
		∆G ^1^	Ki ^2^	∆G	Ki
*E. coli*	4AKE	−5.82	54.17	−6.65	13.26
	3HPQ	−7.24	4.97	−7.27	4.68
*S. pneumoniae*	4NTZ	−1.91	37.12	−7.33	4.22
	4NT0	−7.13	5.92	−7.74	2.13

^1^ ∆G (kcal/mol); ^2^ Ki (µM).

## Supplementary Materials

The following are available online: Figure S1: AKs in closed conformations docked with derivative **1c**; Figure S2: AKs in closed conformation docked with derivative **1d**; Table S1: Comparison of the binding energy ∆G (kcal/mol) and of the inhibition constant Ki (µM**)** of the closed-conformation AKs, Table S2: The interactions of **1c** with selected AKs; Table S3: The interactions of **1d** with selected AKs; Table S4: The characterization of 5-arylidene-2-thioxothiazolidin-4-one derivatives; Table S5: The main characteristics of the AKs used in protein-ligand docking.
