# Supplementary material for: Molecular Docking Evaluation of (E)-5-arylidene-2-thioxothiazolidin-4-one Derivatives as Selective Bacterial Adenylate Kinase Inhibitors
Source: Molecules. 2018 May 3;23(5):1076. doi: 10.3390/molecules23051076 (PMC6102543; doi:10.3390/molecules23051076)
Supplement: Supplementary file 1 [file molecules-23-01076-s001.zip › molecules-289356-SI/Supplementary Materials/Figure S1.doc]

(a) 3HPQ *E.coli.*

Code color for interactions: in green are shown conventional hydrogen bonds, yellow – carbon hydrogen bonds, orange – Sulfur-X, red – unfavorable acceptor-acceptor, violet – Pi-sigma, pink – alkyl/Pi-alkyl, blue – halogen (Cl, Br, I).

(b) 4NU0 *S.pneumoniae*

Code color for interactions: in green are shown conventional hydrogen bonds, yellow – carbon hydrogen bonds, orange – Sulfur-X, red – unfavorable acceptor-acceptor, violet – Pi-sigma, pink – alkyl/Pi-alkyl, blue – halogen (Cl, Br, I).

(c) 1P3J *B.subtilis*

(d) 1ZIP *B. stearothermophilus*

Code color for interactions: in green are shown conventional hydrogen bonds, yellow – carbon hydrogen bonds, orange – Sulfur-X, red – unfavorable acceptor-acceptor, violet – Pi-sigma, pink – alkyl/Pi-alkyl, blue – halogen (Cl, Br, I).

(e) 1S3G *B.globisporus*

(f) 2RGX *A.aeolicus*

Code color for interactions: in green are shown conventional hydrogen bonds, yellow – carbon hydrogen bonds, orange – Sulfur-X, red – unfavorable acceptor-acceptor, violet – Pi-sigma, pink – alkyl/Pi-alkyl, blue – halogen (Cl, Br, I).

(g) 1KHT chain B *M.voltae*

(h) 1NKS chain F *S.acidocaldarius*

**Figure S1.** AKs in closed conformations docked with **1c** derivative. **(a)** 3HPQ *E.coli* **(b)** 4NU0 *S.pneumoniae* **(c)** 1P3J *B.subtilis* **(d)** 1ZIP *B. stearothermophilus* **(e)** 1S3G *B.globisporus* **(f)** 2RGX *A.aeolicus* **(g)** 1KHT chain B *M.voltae*. **(h)** 1NKS chain F *S.acidocaldarius*

Code color for interactions: in green are shown conventional hydrogen bonds, yellow – carbon hydrogen bonds, orange – Sulfur-X, red – unfavorable acceptor-acceptor, violet – Pi-sigma, pink – alkyl/Pi-alkyl, blue – halogen (Cl, Br, I).
