# Supplementary material for: Molecular Docking Evaluation of (E)-5-arylidene-2-thioxothiazolidin-4-one Derivatives as Selective Bacterial Adenylate Kinase Inhibitors
Source: Molecules. 2018 May 3;23(5):1076. doi: 10.3390/molecules23051076 (PMC6102543; doi:10.3390/molecules23051076)
Supplement: Supplementary file 1 [file molecules-23-01076-s001.zip › molecules-289356-SI/Supplementary Materials/Table S1.doc]

**Table S1. Comparison of the binding energy ∆G (kcal/mol) and of the inhibition constant Ki (µM) of the closed-conformation AKs**

| **AKs** → | **Bacteria^2^** | | | **Eukarya (human)^3^** | | **Archaea^4^** | | | |
| --- | --- | --- | --- | --- | --- | --- | --- | --- | --- |
| **derivatives**↓ | **∆G^1^ (kcal/mol)** | | **Ki^1^ (µM)** | **∆G^1^ (kcal/mol)** | **Ki^1^ (µM)** | **∆G^1^ (kcal/mol)** | **Ki^1^ (µM)** | | |
| 1a | -6.49 (±0.52) | | 26.10 (±31.56) | -6.46 (±0.68) | 32.76 (±42.62) | -6.46 (±0.59) | | 47.99 (±15.79) |  |
| 1b | -6.84 (±0.38) | | 11.54 (±8.56) | -5.91 (±0.43) | 58.17 (±46.90) | -6.55 (±0.61) | | 20.22 (±17.83) |  |
| **1c** | **-7.10 (±0.15)** | | **6.46 (±1.90)** | -6.14 (±0.50) | 40.23 (±36.46) | **-7.01 (±0.35)** | | **7.81 (±4.33)** |  |
| 1d | -6.94 (±0.56) | | 11.12 (±8.41) | -6.28 (±0.52) | 32.42 (±26.58) | -6.70 (±0.01) | | 12.21 (±0.27) |  |
| 1e | -6.82 (±0.5) | | 13.60 (±12.77) | -6.00 (±0.68) | 71.78 (±92.62) | -6.83 (±0.20) | | 10.20 (±3.36) |  |
| 1f | -6.62 (±0.26) | | 15.19 (±7.82) | -6.30 (±0.68) | 42.59 (±56.14) | -6.46 (±0.06) | | 18.38 (±1.81) |  |
| ∆G(kcal/mol) | | *f-ratio* value = 13.91418 at p-value = 0.000383 | | | | | | |  |
|  |  | H statistic = 11.9415 at p-value = 0.00255 | | | | | | |  |
| Ki (µM) | | *f-ratio* = 10.69774 at p-value = 0.001297 | | | | | | |  |
|  |  | H statistic = 9.2749 at p-value = 0.00968 | | | | | | |  |

^1^ ∆G and Ki were expressed as Mean (±Standard Deviation); ^2^PDB ID: 2RGX, 3HPQ, 4NT0, 1P3J, 1ZIP, 1S3G; ^3^PDB ID: 1Z83, 2C95, 2C9Y, 2BBW, 2BWJ/chain B; ^4^PDB ID: 1KHT/chain B, 1NKS/chain F.
