# Supplementary material for: Molecular Docking Evaluation of (E)-5-arylidene-2-thioxothiazolidin-4-one Derivatives as Selective Bacterial Adenylate Kinase Inhibitors
Source: Molecules. 2018 May 3;23(5):1076. doi: 10.3390/molecules23051076 (PMC6102543; doi:10.3390/molecules23051076)
Supplement: Supplementary file 1 [file molecules-23-01076-s001.zip › molecules-289356-SI/Supplementary Materials/Table S2.doc]

**Table S2.** The interactions of **1c** with selected AKs

| **AK**  **PDB ID** | **Interactions^1^** | | | | **Distance**  **(Å)** | | | | **∆G (kcal/mol)** | | **Ki (µM)** | | |
| --- | --- | --- | --- | --- | --- | --- | --- | --- | --- | --- | --- | --- | --- |
|  | Hydrogen bonds | | |  | | | |  | | |  | |  |
| **3HPQ** | Gly10 N : O  Ala11 N : O  Gly12 N : O  Lys13 NZ : O  Arg123 NH1 : S | | | 3.39  2.83  2.95  2.63  3.67 | | | | -7.24 | | | 4.97 | |  |
|  | van der Waals | | |  | | | |  | | |  | |  |
|  | Ala8  Thr15  Asp84  Arg119  Ile120 | | |  | | | |  | | |  | |  |
|  | Halogen bonds | | |  | | | |  | | |  | |  |
|  | Tyr133 O : Br | | | 2.87 | | | |  | | |  | |  |
|  | Carbon-hydrogen bonds | | |  | | | |  | | |  | |  |
|  | His134 CD2 : Br | | | 3.32 | | | |  | | |  | |  |
|  | Pi-donor hydrogen bonds | | | | | | | | | | | |  |
|  | Gly14 N |  |  | | | 3.36 |  | | |  | |  | |
|  | (Electrostatic) Pi-Cation | | |  | | | |  | | |  | |  |
|  | Arg123 NH1 | | | 4.34 | | | |  | | |  | |  |
|  | (Hydrophobic) Alkyl or Pi-Alkyl | | | | | | |  | | |  | |  |
|  | Lys13 : Br  His134 : Br  Arg123 : Br  Arg123  Val132  Val132 | | | 4.49  4.32  3.66  4.90  5.25  5.01 | | | |  | | |  | |  |
|  | Hydrogen bonds | | |  | | | |  | | |  | |  |
| **4NU0** | Gly12 HN : O  Lys13 HN : O  Arg128 HH : S  Arg167 HH21: S | | | 2.77  2.09  2.54  2.77 | | | | -7.13 | | | 5.92 | |  |
|  | van der Waals | | |  | | | |  | | |  | |  |
|  | Leu8  Pro9  Gly14  Thr15  Arg89  Arg124  Thr137  Arg156 | | |  | | | |  | | |  | |  |
|  | Pi-Donor Hydrogen | | |  | | | |  | | |  | |  |
|  | Gly 10 HN | | | 2.31 | | | |  | | |  | |  |
|  | Halogen bonds | | |  | | | |  | | |  | |  |
|  | Phe139 O : Br | | | 3.26 | | | |  | | |  | |  |
|  | (Electrostatic) Pi-Cation | | |  | | | |  | | |  | |  |
|  | Arg128 NH1 : N | | | 3.82 | | | |  | | |  | |  |
|  | (Hydrophobic) Alkyl or Pi-Alkyl | | | | | | |  | | |  | |  |
|  | Lys13  Arg128 : Br  Arg128  His139 : Br | | | 4.98  3.70  5.40  5.37 | | | |  | | |  | |  |
|  | Unfavorable Donor-Donor (Acceptor/Donor Clash) | | | | | | | | | |  | |  |
|  | Lys13 HZ3 : N | | | 2.58 | | | |  | | |  | |  |
|  | Hydrogen bonds | | |  | | | |  | | |  | |  |
| **1P3J** | Ala11 N : O  Gly12 N : O  Lys13 NZ : O  Arg127 NH2 : S | | | 3.28  3.22  2.61  3.38 | | | | -7.08 | | | 6.49 | |  |
|  | van der Waals | | |  | | | |  | | |  | |  |
|  | Leu8  Pro9  Gly10  Thr15  Ser30  Asp84  Thr136 | | |  | | | |  | | |  | |  |
|  | Pi-Donor Hydrogen | | |  | | | |  | | |  | |  |
|  | Gly14 N | | | 3.59 | | | |  | | |  | |  |
|  | (Electrostatic) Pi-Cation | | |  | | | |  | | |  | |  |
|  | Arg127 NH2 | | | 4.22 | | | |  | | |  | |  |
|  | Halogen bonds | | |  | | | |  | | |  | |  |
|  | Tyr137 O: Br | | | 3.22 | | | |  | | |  | |  |
|  | (Hydrophobic) Alkyl or Pi-Alkyl | | | | | | |  | | |  | |  |
|  | Arg127  His138 : Br  Lys13  Arg123  Arg127 | | | 3.99  4.48  4.63  5.18  5.01 | | | |  | | |  | |  |
|  | Hydrogen bonds | | |  | | | |  | | |  | |  |
| **1ZIP** | Gly12 N : O  Lys13 N : O  Lys13 NZ : O  Arg127 NH1 : S  Arg171 NH2 : S | | | 3.11  2.77  2.89  3.14  3.17 | | | | -6.43 | | | 6.39 | |  |
|  | van der Waals | | |  | | | |  | | |  | |  |
|  | Pro9  Gly10  Ala11  Gly14  Thr15  Arg123  Leu124  Thr136  Tyr137  Arg160 | | |  | | | |  | | |  | |  |
|  | Carbon-hydrogen bonds | | |  | | | |  | | |  | |  |
|  | His138 CD2 : Br | | | 3.58 | | | |  | | |  | |  |
|  | Pi-Donor Hydrogen | | |  | | | |  | | |  | |  |
|  | Gly10 N : | | | 2.99 | | | |  | | |  | |  |
|  | (Electrostatic) Pi-Cation | | |  | | | |  | | |  | |  |
|  | Arg127 NH1 | | | 3.73 | | | |  | | |  | |  |
|  | (Hydrophobic) Alkyl or Pi-Alkyl | | | | | | |  | | |  | |  |
|  | Arg127 : Br  His 138 : Br  Lys13  Arg127 | | | 3.64  4.65  5.16  5.22 | | | |  | | |  | |  |
|  | Unfavorable Donor-Donor (Acceptor/Donor Clash) | | | | | | | | | |  | |  |
|  | Lys13 NZ : N | | | 2.71 | | | |  | | |  | |  |
|  | Hydrogen bonds | | |  | | | |  | | |  | |  |
| **1S3G** | Gly10 N : O | | | 2.99 | | | | -6.82 | | | 10.09 | |  |
|  | van der Waals | | |  | | | |  | | |  | |  |
|  | Leu8  Pro9  Ala11  Gly12  Thr15  Asp84  Arg123  Leu124  Ser136  Tyr137 | | |  | | | |  | | |  | |  |
|  | (Electrostatic) Pi-Cation/ Pi-Donor | | | | | | | | | |  | |  |
|  | Arg127 NH2 | | | 3.78 | | | |  | | |  | |  |
|  | Pi-Donor Hydrogen | | |  | | | |  | | |  | |  |
|  | Gly14 N  Arg127 NE | | | 3.66  3.96 | | | |  | | |  | |  |
|  | (Hydrophobic) Alkyl, Pi-Alkyl, or Pi-sigma | | | | | | | | | |  | |  |
|  | Lys13 CB  Arg127 : Br  His138 : Br  Arg127 | | | 3.85  4.20  5.04  5.23 | | | |  | | |  | |  |
|  | Hydrogen bonds | | |  | | | |  | | |  | |  |
| **2RGX** | Thr31 OG1 : S  Gln89 NE2 : O | | | 3.11  3.29 | | | | -7.24 | | | 4.93 | |  |
|  | van der Waals | | |  | | | |  | | |  | |  |
|  | Leu35  Gly56  Glu57  Gly82 | | |  | | | |  | | |  | |  |
|  | Halogen bonds | | |  | | | |  | | |  | |  |
|  | Asp152 OD2 : Br | | | 3.26 | | | |  | | |  | |  |
|  | Carbon-hydrogen bonds | | |  | | | |  | | |  | |  |
|  | Arg161 CD : Br | | | 3.72 | | | |  | | |  | |  |
|  | Pi-Sulfur | | |  | | | |  | | |  | |  |
|  | Phe83 | | | 4.48 | | | |  | | |  | |  |
|  | (Hydrophobic) Alkyl, Pi-Alkyl, or Pi-sigma | | | | | | | | | |  | |  |
|  | Ile64 CD1  Met53 : Br  Arg161 : Br  Leu58  Val59  Arg85  Met53 | | | 3.54  4.94  5.15  5.00  5.33  4.02  5.25 | | | |  | | |  | |  |
|  | Unfavorable Donor-Donor (Acceptor/Donor Clash) | | | | | | | | | |  | |  |
|  | Gln89 NE2 : N | | | 2.86 | | | |  | | |  | |  |
|  | Hydrogen bonds | | |  | | | |  | | |  | |  |
| **1KHT**/chainB | Asp57 OD2 : N  Arg60 NE : N  Met59 N : O  Arg138 NH2 : S | | | 2.79  2.94  3.08  3.29 | | | | -7.26 | | | 4.74 | |  |
|  | van der Waals | | |  | | | |  | | |  | |  |
|  | Pro12  Arg46  Gln58  Gln67  Gly104 | | |  | | | |  | | |  | |  |
|  | Halogen bonds | | |  | | | |  | | |  | |  |
|  | Gln71 OE1: Br14 | | | 3.14 | | | |  | | |  | |  |
|  | (Hydrophobic) Alkyl, Pi-Alkyl, or Pi-sigma | | | | | | | | | |  | |  |
|  | Met43 CE  Val95 CG2  Met43 : Br  Val95 : Br  Leu102 : Br  Arg60 | | | 3.95  3.53  5.18  4.97  5.11  4.08 | | | |  | | |  | |  |
|  | Unfavorable Donor-Donor (Acceptor/Donor Clash) | | | | | | | | | |  | |  |
|  | Arg60 NE : N | | | 2.94 | | | |  | | |  | |  |
| **1NKS**/chainF | Hydrogen bonds | | |  | | | | -6.77 | | | 10.87 | |  |
|  | Arg141 NH1 : O  Tyr144 OH : N | | | 2.88  2.65 | | | |  | | |  | |  |
|  | van der Waals | | |  | | | |  | | |  | |  |
|  | Gly38  Val95  Thr153 | | |  | | | |  | | |  | |  |
|  | Pi-Donor Hydrogen | | |  | | | |  | | |  | |  |
|  | Arg97 N | | | 3.72 | | | |  | | |  | |  |
|  | Halogen bonds | | |  | | | |  | | |  | |  |
|  | Met41 SD : Br | | | 3.62 | | | |  | | |  | |  |
|  | Sulfur bonds | | |  | | | |  | | |  | |  |
|  | Tyr144 F : S | | | 5.74 | | | |  | | |  | |  |
|  | (Hydrophobic) Alkyl, Pi-Alkyl, Pi-sigma, or Pi-Pi Stacked | | | | | | | | | |  | |  |
|  | His93  Met41 : Br  Met57  Pro10  Ile96  Arg97  Arg58  Ile96 | | | 4.61  3.94  4.32  4.26  5.16  4.57  4.96  4.96 | | | |  | | |  | |  |

^1^ blue: residues belong to ATP lid; red: residues belong to AMP bd
