# Supplementary material for: Molecular Docking Evaluation of (E)-5-arylidene-2-thioxothiazolidin-4-one Derivatives as Selective Bacterial Adenylate Kinase Inhibitors
Source: Molecules. 2018 May 3;23(5):1076. doi: 10.3390/molecules23051076 (PMC6102543; doi:10.3390/molecules23051076)
Supplement: Supplementary file 1 [file molecules-23-01076-s001.zip › molecules-289356-SI/Supplementary Materials/Table S3.doc]

**Table S3.** The interactions of **1d** with selected AKs

| **AK**  **PDB ID** | **Interactions^1^** | | | | **Distance**  **(Å)** | | | | **∆G (kcal/mol)** | | **Ki (µM)** | | |
| --- | --- | --- | --- | --- | --- | --- | --- | --- | --- | --- | --- | --- | --- |
|  | Hydrogen bonds | | |  | | | |  | | |  | |  |
| **3HPQ** | Thr 15 N : O  Thr 15 OG1 : O | | | 2.78  2.91 | | | | -7.27 | | | 4.68 | |  |
|  | van der Waals | | |  | | | |  | | |  | |  |
|  | Gly 10  Ala 11  Gly 12  Asp 84  Ile 120  Val 132  His 134 | | |  | | | |  | | |  | |  |
|  | Unfavorable Donor-Donor (Acceptor/Donor Clash) | | | | | | | | | | | |  |
|  | Gly 14 N : N |  |  | | | 2.73 |  | | |  | |  | |
|  | (Electrostatic) Pi-Cation | | |  | | | |  | | |  | |  |
|  | Arg 123 NH1 | | | 4.00 | | | |  | | |  | |  |
|  | (Hydrophobic) Alkyl or Pi-Alkyl | | | | | | |  | | |  | |  |
|  | Lys 13  Arg 119  Arg 123 | | | 5.29  4.26  4.75 | | | |  | | |  | |  |
|  | Hydrogen bonds | | |  | | | |  | | |  | |  |
| **4NU0** | Gly 7 HN : O  Lys 13 HZ1 : O | | | 2.21  2.32 | | | | -7.74 | | | 2.13 | |  |
|  | van der Waals | | |  | | | |  | | |  | |  |
|  | Leu 4  Ile 5  Pro 9  Leu 84  Asp 85  Gly 86  Pro 88  Tyr 87  Gly 175  Ile 179 | | |  | | | |  | | |  | |  |
|  | Carbon Hydrogen bonds | | |  | | | |  | | |  | |  |
|  | Lys 13 HE2 : O | | | 2.71 | | | |  | | |  | |  |
|  | Halogen bonds | | |  | | | |  | | |  | |  |
|  | Asn 171 OD1 : Br | | | 3.22 | | | |  | | |  | |  |
|  | Sulfur X or Pi-Sulfur | | |  | | | |  | | |  | |  |
|  | Met 6 SD : S  Met 6 SD | | | 3.32  5.32 | | | |  | | |  | |  |
|  | (Hydrophobic) Alkyl or Pi-Alkyl | | | | | | |  | | |  | |  |
|  | Met 6  Leu 8  Arg 89 : Br | | | 3.84  5.48  4.94 | | | |  | | |  | |  |
|  | Unfavorable Donor-Donor (Acceptor/Donor Clash) | | | | | | | | | |  | |  |
|  | Gly 7 O : O14 | | | 2.83 | | | |  | | |  | |  |
|  | Hydrogen bonds | | |  | | | |  | | |  | |  |
| **1P3J** | Val 59 N : O  Gly 85 O : S  Gln 92 NE2 : S | | | 2.69  3.17  3.55 | | | | -7.25 | | | 4.83 | |  |
|  | van der Waals | | |  | | | |  | | |  | |  |
|  | Thr 31  Gly 32  Arg 36  Gly 56  Thr 64  Arg 160  Asp 162  Arg 171 | | |  | | | |  | | |  | |  |
|  | (Electrostatic) Pi-Cation | | |  | | | |  | | |  | |  |
|  | Arg 88 NH1 | | | 4.47 | | | |  | | |  | |  |
|  | Pi-Sulfur | | |  | | | |  | | |  | |  |
|  | Phe 35 : S  Phe 86 : S | | | 4.70  5.30 | | | |  | | |  | |  |
|  | (Hydrophobic) Alkyl or Pi-Alkyl | | | | | | |  | | |  | |  |
|  | Phe 35  Ile 53 CG2  Val 59  Arg 88 | | | 4.96  3.89  5.14  5.46 | | | |  | | |  | |  |
|  | Hydrogen bonds | | |  | | | |  | | |  | |  |
| **1ZIP** | Ala 11 N : O  Lys 13 NZ : O | | | 3.15  3.62 | | | | -7.25 | | | 19.21 | |  |
|  | van der Waals | | |  | | | |  | | |  | |  |
|  | Leu 8  Gly 10  Ser 30  Gly 32  Asp 33  Arg 36  Asp 84  Arg 127  Arg 160  Arg 171 | | |  | | | |  | | |  | |  |
|  | Pi-donor Hydrogen | | |  | | | |  | | |  | |  |
|  | Gly 14 N | | | 4.04 | | | |  | | |  | |  |
|  | Pi-Cation  Arg 88 | | |  | | | |  | | |  | |  |
|  | (Electrostatic) Pi-Cation | | |  | | | |  | | |  | |  |
|  | Arg 88 NH2 | | | 4.65 | | | |  | | |  | |  |
|  | (Hydrophobic) Alkyl or Pi-Alkyl | | | | | | |  | | |  | |  |
|  | Pro 9 : Br  Lys 13 | | | 4.84  5.04 | | | |  | | |  | |  |
|  | Unfavorable Donor-Donor (Acceptor/Donor Clash) | | | | | | | | | |  | |  |
|  | Gly 12 N : N | | | 2.84 | | | |  | | |  | |  |
|  | Hydrogen bonds | | |  | | | |  | | |  | |  |
| **2RGX** | Val 59 N: O  Gln 89 OE1 : S | | | 2.75  3.00 | | | | -6.63 | | | 13.87 | |  |
|  | van der Waals | | |  | | | |  | | |  | |  |
|  | Thr 31  Gly 32  Arg 36  Leu 35  Gly 56  Glu 57  Gly 82  Gly 84 | | |  | | | |  | | |  | |  |
|  | Pi-cation | | |  | | | |  | | |  | |  |
|  | Arg 85 NH1 | | | 4.91 | | | |  | | |  | |  |
|  | Pi-Sulfur | | |  | | | |  | | |  | |  |
|  | Phe 83 : S | | | 4.59 | | | |  | | |  | |  |
|  | (Hydrophobic) Alkyl, Pi-Alkyl, or Pi-sigma | | | | | | | | | |  | |  |
|  | Met 53 CG  Ile 64 CD1  Leu 58 : Br  Arg 161 : Br  Leu 58  Val 59  Arg 85 | | | 3.86  3.86  5.48  4.53  5.02  5.47  4.00 | | | |  | | |  | |  |
|  | Hydrogen bonds | | |  | | | |  | | |  | |  |
| **1KHT**/chainB | Gly 40 N : O  Thr 91 OG1 : O  Thr 91 OG1 : N  Glu 71 OE1 : S  Gly 104 O : S | | | 2.40  2.91  2.78  3.18  3.34 | | | | -6.71 | | | 12.02 | |  |
|  | van der Waals  Phe 39  Phe 44  Asp 57  His 92 | | |  | | | |  | | |  | |  |
|  | (Electrostatic) Pi-Cation | | |  | | | |  | | |  | |  |
|  | Arg 138 NH2 | | | 4.71 | | | |  | | |  | |  |
|  | (Hydrophobic) Alkyl, Pi-Alkyl, or Pi-sigma | | | | | | | | | |  | |  |
|  | Arg 56 : Br  Met 59 : Br  Arg 60  Val 95  Val 95 | | | 4.03  4.22  5.11  5.41  5.27 | | | |  | | |  | |  |
|  | Pi- Sulfur | | | | | | | | | |  | |  |
|  | Met 43 SD : S | | | 3.91 | | | |  | | |  | |  |

^1^ blue: residues belong to ATP lid; red: residues belong to AMP bd
