# Supplementary material for: Molecular Docking Evaluation of (E)-5-arylidene-2-thioxothiazolidin-4-one Derivatives as Selective Bacterial Adenylate Kinase Inhibitors
Source: Molecules. 2018 May 3;23(5):1076. doi: 10.3390/molecules23051076 (PMC6102543; doi:10.3390/molecules23051076)
Supplement: Supplementary file 1 [file molecules-23-01076-s001.zip › molecules-289356-SI/Supplementary Materials/Table S4.doc]

**Table S4.** The characterisation of the 5-arylidene-2-thioxothiazolidin-4-one derivatives

| **derivatives** | **IUPAC name** | **Formula** | **Molecular weight** |
| --- | --- | --- | --- |
| **1a** | 5-(3-methoxybenzylidene)-2-thioxothiazolidin-4-one | C_11_H_17_NO_2_S_2_ | 259.38 |
| **1b** | 5-(4-chlorobenzylidene)-2-thioxothiazolidin-4-one | C_10_H_12_ClNOS_2_ | 261.78 |
| **1c** | 5-(4-bromobenzylidene)-2-thioxothiazolidin-4-one | C_10_H_12_BrNOS_2_ | 306.24 |
| **1d** | 5-(2-bromobenzylidene)-2-thioxothiazolidin-4-one | C_10_H_12_BrNOS_2_ | 306.24 |
| **1e** | 5-(3-chlorobenzylidene)-2-thioxothiazolidin-4-one | C_10_H_12_ClNOS_2_ | 261.78 |
| **1f** | 5-(2-methoxybenzylidene)-2-thioxothiazolidin-4-one | C_11_H_17_NO_2_S_2_ | 259.38 |
