# Supplementary material for: Molecular Docking Evaluation of (E)-5-arylidene-2-thioxothiazolidin-4-one Derivatives as Selective Bacterial Adenylate Kinase Inhibitors
Source: Molecules. 2018 May 3;23(5):1076. doi: 10.3390/molecules23051076 (PMC6102543; doi:10.3390/molecules23051076)
Supplement: Supplementary file 1 [file molecules-23-01076-s001.zip › molecules-289356-SI/Supplementary Materials/Table S5.doc]

**Table S5** The main characteristics of the AKs used in protein-ligand docking

| ACCESSION NUMBER | Source  (tested chains) | co-crystallized with inhibitor or other substances | No. of  residues | Ref. |
| --- | --- | --- | --- | --- |
|  | *Bacterial source* | | | |
| 3HPQ | *E.coli* | P1,P5-di(adenosine-5')pentaphosphate  (Ap5A) | 214 | [36] |
| 4AKE | *E.coli* | no inhibitors/other substances | 214 | [23] |
| 4NTZ | *S.pneumoniae* | SO_4_^2-^ | 217 | [37] |
| 4NU0 | *S.pneumoniae* | Ap5A, Mg^2+^ | 217 | [37] |
| 1ZIP | *B. stearothermophilus* | Ap5A, Mn^2+^, and Mg^2+^ | 217 | [38] |
| 1P3J | *B.subtilis* | Ap5A, Mg^2+^, and Zn^2+^ | 217 | [39] |
| 1S3G | *B.globisporus* | Ap5A and Zn^2+^ | 217 | [39] |
| 2RH5 | *A.aeolicus* [(strain VF5)](https://www.rcsb.org/pdb/search/smartSubquery.do?smartSearchSubtype=TreeEntityQuery&t=1&n=224324) | no inhibitors/other substances | 206 | [40] |
| 2RGX | *A.aeolicus* [(strain VF5)](https://www.rcsb.org/pdb/search/smartSubquery.do?smartSearchSubtype=TreeEntityQuery&t=1&n=224324) | Ap5A and Zn^2+^ | 206 | [40] |
|  | *Homo sapiens* | | | |
| 2C95 | AK1  (tested chain A) | P1,P4-Di(Adenosine)tetraphosphate; malonate ion (chains A,B) | 196 | Unpub |
| 1Z83 | AK1A  (tested chain A) | Bis(adenosine)-5’-pentaphosphate Zn^2+^ , and SO_4_^2-^ (chains A,B, C) | 195 | Unpub |
| 2C9Y | AK2  (tested chain A) | Bis(Adenosine)-5'-Tetraphosphate, Ethylene Glycol (chain A) | 242 | Unpub |
| 2AR7 | AK4  (tested chain A) | no inhibitors/other substances  (chains A, B) | 246 | Unpub |
| 2BBW | AK4  (tested chains A) | **Diguanosine-pentaphosphate**  (chains A, B) | 246 | Unpub |
| 2BWJ | AK5  (tested chains A, B) | AMP (chains B, C, F)  Cl^-^ (chains A, B, E, F)  SO_4_^2-^ (chains A, B, C, D, E, F) | 199 | Unpub |
|  | *Archaea source* | | | |
| 1NKS | *S.acidocaldarius*  (tested chains A, C, F) | AMP (chain C, D, F)  adenosine 5’-diphosphate (chain F) | 194  (trimeric) | [42] |
| 1KHT | *M.voltae*  (tested chains A, B) | (adenosine monophosphate) AMP  (chains B, C) | 192  (trimeric | [41] |

***References***

23.	Müller, C.W.; Schlauderer, G.J.; Reinstein, J.; Schulz, G.E. Adenylate kinase motions during catalysis: an energetic counterweight balancing substrate binding. *Structure.* **1996,** 4, 147–156.

36.	Schrank, T.P.; Bolen, D.W.; Hilser, V.J. Rational modulation of conformational fluctuations in adenylate kinase reveals a local unfolding mechanism for allostery and functional adaptation in proteins. *Proc. Natl. Acad. Sci. U. S. A.* **2009**, *106*, 16984–16989. Available from: 10.1073/pnas.090651010

37.	Thach, T.T.; Luong, T.T.; Lee, S.; Rhee, D.K. Adenylate kinase from Streptococcus pneumoniae is essential for growth through its catalytic activity. FEBS Open Bio [Internet] **2014**, *4*, 672–862. DOI: 10.1016/j.fob.2014.07.002

38.	Berry, M.B.; Phillips, G.N.Jr. Crystal structures of Bacillus stearothermophilus adenylate kinase with bound Ap5A, Mg2+ Ap5A, and Mn2+ Ap5A reveal an intermediate lid position and six coordinate octahedral geometry for bound Mg2+ and Mn2+. *Proteins,* **1998**, *32*, 276–288.

39.	Bae, E.; Phillips, G.N. Structures and analysis of highly homologous psychrophilic, mesophilic, and thermophilic adenylate kinases. *J. Biol. Chem*. **2004,** *279*, 28202–28208. DOI: 10.1074/jbc.M401865200

40.	Henzler-Wildman, K.A.; Thai, V.; Lei, M.; Ott, M.; Wolf-Watz, M.; Fenn, T.; Pozharski, E.; Wilson, M.A.; Petsko, G.A.; Karplus, M.; Hübner, C.G.; Kern, D. Intrinsic motions along an enzymatic reaction trajectory. *Nature* **2007,** *450*, 838–844. DOI: DOI:10.1038/nature06410

41.	Criswell, A.R.; Bae, E.; Stec, B.; Konisky, J.; Phillips, G.N. Jr. Structures of Thermophilic and Mesophilic Adenylate Kinases from the Genus Methanococcus. *J. Mol. Biol.* **2003**, *330*, 1087–1099.

42.	Vonrhein, C.; Bönisch, H.; Schäfer, G.; Schulz, G.E. The structure of a trimeric archaeal adenylate kinase. *J. Mol. Biol*. **1998,** *282*, 167–79.
